# Compensatory expression of NRF2-dependent antioxidant genes is required to overcome the lethal effects of Kv11.1 activation in breast cancer cells and PDOs

**DOI:** 10.1016/j.redox.2021.102030

**Published:** 2021-06-12

**Authors:** Vitalyi Senyuk, Najmeh Eskandari, Ying Jiang, Rebeca Garcia-Varela, Rachel Sundstrom, Luigi Leanza, Roberta Peruzzo, Mark Burkard, Richard D. Minshall, Saverio Gentile

**Affiliations:** a Division of Hematology Oncology, Department of Medicine, University of Illinois Chicago, Chicago, IL, USA; b Departments of Anesthesiology and Pharmacology and Regenerative Medicine, University of Illinois, Chicago, IL, USA; c Departments of Oncology and Medicine, Hematology and Oncology, and the UW Carbone Cancer Center, University of Wisconsin—Madison, Madison, WI, USA; d Department of Biology, University of Padova, Padova, Italy; e Tecnologico de Monterrey, Centro de Biotecnologia-FEMSA, Escuela de Ingenieria y Ciencias, Monterrey N.L., Mexico

**Keywords:** NRF2, Potassium channels, Mitochondria, Cancer cell survival

## Abstract

Potassium channels are important regulators of cellular homeostasis and targeting these proteins pharmacologically is unveiling important mechanisms in cancer cell biology. Here we demonstrate that pharmacological stimulation of the Kv11.1 potassium channel activity results in mitochondrial reactive oxygen species (ROS) production and fragmentation in breast cancer cell lines and patient-derived organoids independent of breast cancer subtype. mRNA expression profiling revealed that Kv11.1 activity significantly altered expression of genes controlling the production of ROS and endoplasmic-reticulum (ER) stress. Characterization of the transcriptional signature of breast cancer cells treated with Kv11.1 potassium channel activators strikingly revealed an adaptive response to the potentially lethal augmentation of ROS by increasing Nrf2-dependent transcription of antioxidant genes. Nrf2 in this context was shown to promote survival in breast cancer, whereas knockdown of Nrf2 lead to Kv11.1-induced cell death. In conclusion, we found that the Kv11.1 channel activity promotes oxidative stress in breast cancer cells and that suppression of the Nrf2-mediated anti-oxidant survival mechanism strongly sensitized breast cancer cells to a lethal effect of pharmacological activation of Kv11.1.

## 1 Introduction

Breast cancer is a heterogeneous disease both biologically and clinically with highly variable outcomes. Different types of breast cancer are commonly characterized by the expression levels of estrogen (ER+) and progesterone (PR+) receptors and/or the proto-oncogene receptor protein tyrosine kinase HER2/*neu* (HER2+) [1]. Breast tumors that lack expression of ER and HER2/*neu* proteins, known as triple negative breast cancer (TNBC), are aggressive [2,3], highly metastatic, and have the worst outcome of all BC subtypes. Breast cancer progression is accompanied by genetic alteration of a multitude of genes which alone or in combination can significantly alter a variety of cellular events [4]. Although several therapies have been developed against breast cancer [5], it remains the second leading cause of cancer-related death in women worldwide, claiming more than 550,000 lives per year. Treatment options are often inadequate due to the difficulty in identifying proteins governing crucial biochemical signaling pathways and lack of approved targeted therapies, particularly for TNBC.

Ion channels are the molecular regulators of ion exchange across membranes of all cells, and they have emerged as important players in cancer biology. Several members of the voltage-gated potassium (Kv) channel family, including Kv11.1, have been identified as potential targets for cancer therapy [6,7]. Anatomically, human Kv11.1 is expressed mostly in the brain and in the heart. These tissues are formed by highly differentiated cells that are primarily non-proliferative. Kv11.1 channels are also found in cancers with different histological characteristics and tissues of origin, including breast cancers [[7], [8], [9], [10], [11], [12]], where Kv11.1 activity controls several functional hallmarks of cancer cells such as proliferation, migration, metabolism and survival. However, the biochemical pathways linking K11.1 to these events remain largely unknown.

Mitochondria are the major source of reactive oxygen species (ROS) as byproduct of the respiratory chain which relies heavily on ion homeostasis. Nevertheless, the interaction among ions and ROS can be bidirectional as ions can regulate ROS production and ROS can control activities of several ion channels [[13], [14], [15]] that can be expressed on both mitochondria and surface cell membranes. Increasing evidence indicate that the cross-talk between ions and ROS can play a major role in both physiological and pathological condition. For example, mitochondrial Ca^2+^ homeostasis is fundamental to generate important metabolic processes however, high mitochondrial Ca^2+^ level can initiate cell death pathways [16,17].

We previously reported that the Kv11.1 potassium channel is expressed in breast cancers independently of their molecular and/or histological characterization [18]. Furthermore, pharmacological stimulation of Kv11.1 results in arresting tumor growth by activation of a cellular senescence phenotype [7,[18], [19], [20], [21]]. Although senescent cells downregulate several oncogenes while significantly upregulating tumor suppressors they are metabolically active. Therefore, in this context, senescent phenotype could be considered as a survival mechanism. In this work, we characterized a cellular signaling mechanism linking Kv11.1 activation-dependent increase in mitochondrial ROS production to a NRF2-dependent antioxidant response that overcomes potentially lethal pharmacological treatment.

## 2 Results

### 2.1 Activation of Kv11.1 alters mitochondria structure

**Fig. 1:**
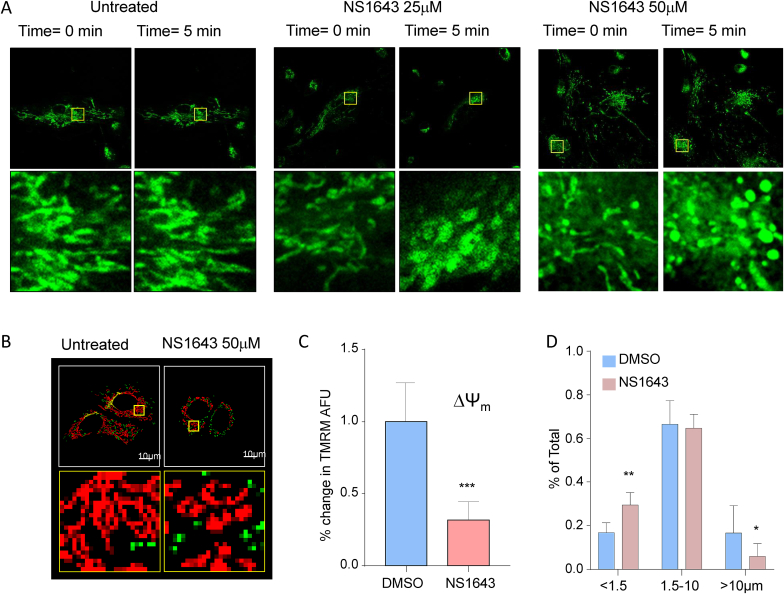
**Activation of Kv11.1 affects mitochondria structure.** A) Mitochondrial morphology highlighted in MDA-MB-231 cells using 200 nM of Mitotracker green for 20 min at 37 °C. Mitochondrial swelling was visible in the NS1643 treated cells 5 min after compound addition. Representative images and magnification of three independent experiments are shown. B) Live cell imaging of MCF7 cells treated with 2 μl DMSO or 50 μM NS1643 before image acquiring. Yellow insert is magnified on the panel below. Pseudo-color masks represent mitochondria in different length group: Green (<1.5 μm), Red (1.5–10 μm) and Yellow (>10 μm). E) Fluorescence intensity of TMRM was measured to represent the change of mitochondrial membrane potential (DΨm). F) Mitochondria categorized into different length groups, the short (<1.5 μm), medium (1.5–10 μm) and elongated (>10 μm). The percentage of each group was calculated by dividing sum area of each group with total mitochondrial area. (For interpretation of the references to color in this figure legend, the reader is referred to the Web version of this article.)

We assessed the impact of Kv11.1 activation on mitochondrial structure. We performed experiments using the mitochondria-specific fluorescent label Mitotracker green (Fig. 1A). Surprisingly, significant mitochondrial swelling was visible in the NS1643 treated cells already 5 min after exposure to different concentrations of the drug. To further characterize this effect, we used Tetramethylrhodamine Methyl Ester (TMRM) fluorescent dye to monitor mitochondria metabolism upon NS1643 application (Fig. 1B). We found that NS1643 produces a strong depolarization (Fig. 1C) of mitochondria ΔΨ_m_ which indicates loss of mitochondria function. Also, live cell imaging revealed that NS1643 treatment shortens the mitochondria (Fig. 1D) confirming the potent effect of Kv11.1 activation on mitochondria morphological structure.

### 2.2 Activation of Kv11.1 alters mitochondria function

**Fig. 2:**
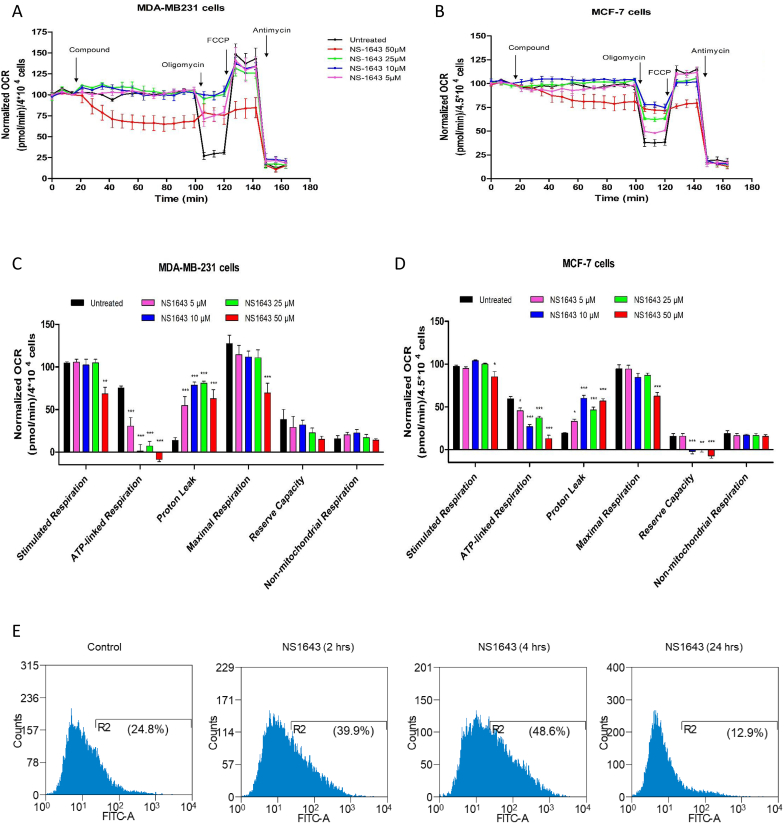
**Activation of Kv11.1 affects mitochondria function.** A) Sea-Horse Analysis of mitochondrial function in TNBC MDA-MB-231 or B) ER + MCF7 breast cancer cells treated with NS1643 reveals the effects on oxygen consumption rate (OCR) of increasing concentrations up to 50 μM of NS1643. Data are show as percentage of the initial value. (n = 3; +/− SEM). Broken lines represent the addition of the indicated compound: NS: NS1643; Oligomycin 2 μg/mL; FCCP: FCCP 500 nM; Antimycin 1 μM. C) Quantification of the NS1643 effects in MDA-MB-231 or D) MCF7 breast cancer cell lines on mitochondria function as indicated. E) Flow cytometry was used to measure ROS production with 2′,7’ –dichlorofluorescin diacetate (DCFDA) dye in MDA-MB-231 breast cancer cells at timepoints indicated.

Next we carried out a Mito Stress Test assay with a Seahorse Analyzer in cells treated with NS1643 or vehicle in human-derived breast cancer cell lines that represents TNBC or ER + cancer subtypes (Fig. 2A; 2B). Interestingly, we found that NS1643 severely effected mitochondria function in a concentration-dependent manner in both TNBC (Fig. 2 A) and ER+ (Fig. 2B) cell lines. We observed an enhanced leak due to NS1643 exposure that is most evident when the cells are uncoupled with the carbonyl cyanide-p-trifluoromethox-phenyl-hydrazon (FCCP), minoxidil-treated cells have a higher oxygen consumption rate (OCR), and a higher leak rate when comparing the oligomycin – Antimycin/Rotenone rates that directly measure leak (Fig. 2A, B, 2C, 2D).

### 2.3 Kv11.1 activity leads to production of reactive oxygen species

Increased flux through the mitochondrial electron transport chain can also increase leak of electrons to oxygen resulting in enhanced formation of ROS. Thus, we monitored the effect of Kv11.1 channel stimulation on ROS production. As expected, we found that cells treated with NS1643 for two or 4 h resulted in significant time-dependent increase of ROS production (Fig. 2E). Surprisingly, we observed a strong reduction of ROS in cells treated for 24hr with NS1643. These results suggest that stimulation of Kv11.1 activity leads to ROS production but ROS accumulation is transient.

### 2.4 Activation of Kv11.1 upregulates antioxidant genes

To better understand our data, we have used a microarray to analyze the mRNA expression profile of human breast cancer cells treated with the Kv11.1 potassium channel activator NS1643. Differentially expressed genes (DEG) were identified between NS1643 treated vs untreated TNBC cell line MDA-MB-231 (Supplementary Figure 1). This analysis revealed 516 genes that were differentially expressed (Benjamini-Hochberg adjusted p-value <0.1 and an absolute log2-fold change of at ≥ 1.5). Among these, 386 were found upregulated and 130 were found to be downregulated by NS1643 treatment.

**Fig. 3:**
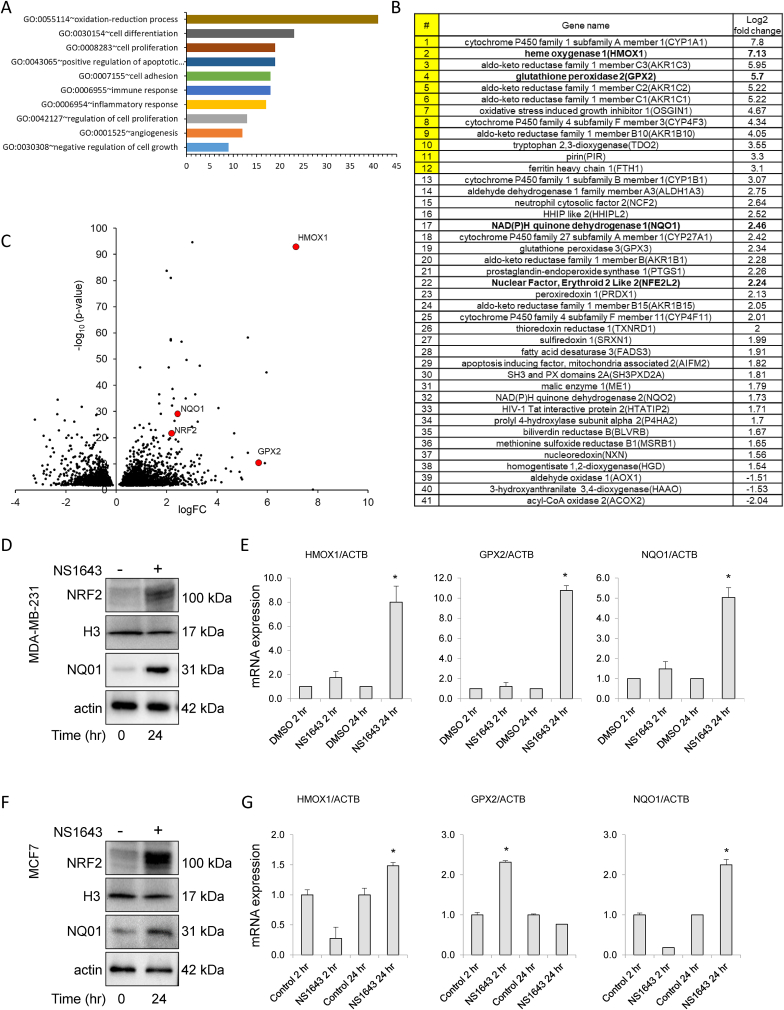
**Kv11.1 potassium channel activity activates oxidative-stress.** A) Analysis of functional gene ontology (GO) of MDA-MD231 breast cancer cells treated with NS1643 (24hr). B) Most differentially expressed genes. Genes from 1 to 12 (yellow) are among the most 30 upregulated genes in the RNA-Seq. C) Distribution of the DEGs shown as volcano plot. Each point represents a gene. The red dots represent genes chosen for validation. D) Western blot and E) RT-QPCR analysis of oxidative stress markers in MDA-MB-231 cells. F) Western blot and G) RT-QPCR analysis of oxidative stress markers in MCF7 cells. (For interpretation of the references to color in this figure legend, the reader is referred to the Web version of this article.)

Next we utilized the data obtained from RNA-Seq analysis to perform a functional analysis using the gene ontology (GO) bioinformatic approach. We found that NS1643 significantly upregulated the cellular oxidation-reduction process (GO:0055114; Fig. 3A; 3B). Remarkably, 12 of the most upregulated anti-oxidant genes were also found in the overall array among the 30 most upregulated genes by NS1643 (Fig. 3B; Supplementary Figure 1). Transcription of these antioxidant genes is regulated by the antioxidant response element (ARE) [22]. Therefore, we hypothesized that ARE-dependent transcription can play a critical role in mediating the antioxidant response upon NS1643 treatment. To test this hypothesis, we validated the in silico analysis by monitoring protein expression and mRNA level of antioxidant genes (Fig. 3C) including heme oxygenase-1 (HMOX1), glutathione peroxidase-2 (GPX2), NAD(P)H dehydrogenase [quinone]-1 (NQO1) and nuclear factor erythroid 2–related factor 2 (NRF2). We found that all redox markers were strongly upregulated in both MDA-MB-231 (Fig. 3D; 3E) and MCF7 (Fig. 3F; 3G) cell lines treated with NS1643 after 24hr with the exception of GPX2 in MCF7 that was upregulated after 2hr (Fig. 3F). In contrast, no significant changes in these markers were recorded in MCF10A after NS1643 treatment at any time (Supplementary Fig. 2A). This data indicates that stimulation of Kv11.1 channel can induce activation of an antioxidant response in breast cancer cells.

### 2.5 NS1643 activates endoplasmic reticulum stress

**Fig. 4:**
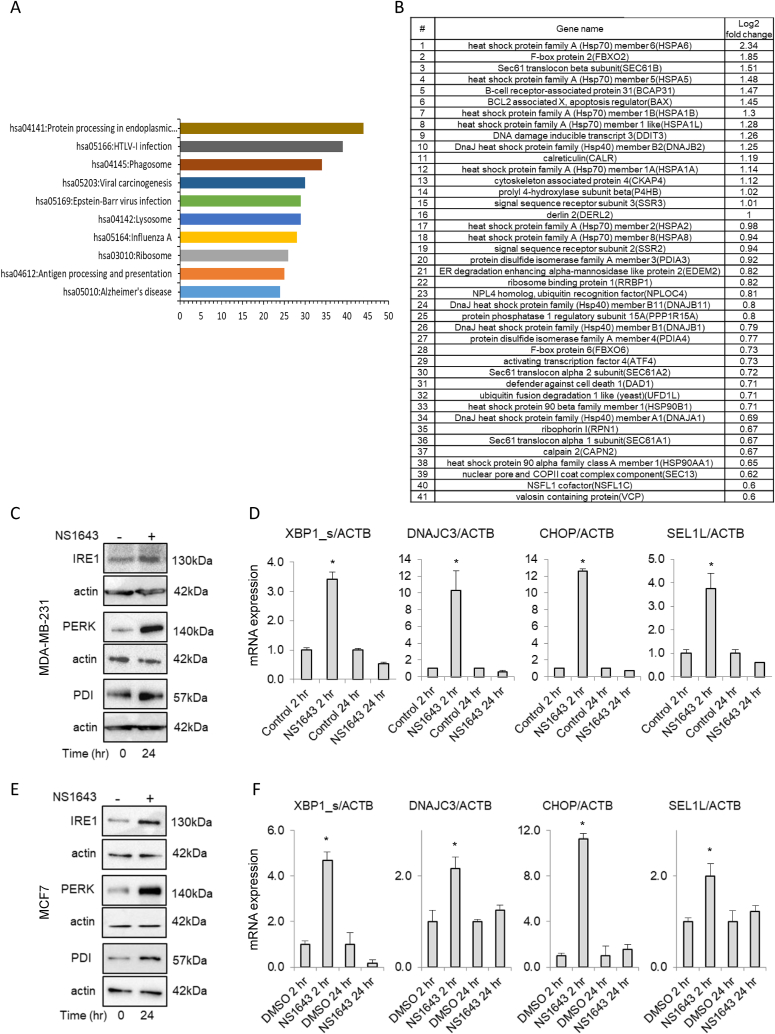
**Kv11.1 potassium channel activation promotes ER-stress.** A) Top 10 biological processes (KEGG Pathway Enrichment Analysis by DAVID) regulated by NS1643 treatment (24hr) of MDA-MB-231 breast cancer cells. B) List of the most differentially expressed genes (DEGs) after NS1643 treatment. C) Western blot and D) RT-QPCR analysis of markers of the unfolded protein response (UPR) in MDA-MB-231 cells. E) Western blot and F) RT-QPCR analysis of UPR markers in MCF7 cells.

Alteration of mitochondria function and ROS production has been associated with endoplasmic reticulum stress (ER-stress). To assess whether stimulation of Kv11.1 could determine ER-stress we first performed analysis of KEGG pathways for DEGs by using DAVID [23] which showed the most significantly upregulated genes associated with protein processing in the endoplasmic reticulum (Fig. 4A; 4B; Supplementary Figure 2B; hsa04141). ER-stress occurs when the capacity of the endoplasmic reticulum to fold proteins becomes saturated. Therefore, to respond to this burden of excess unfolded protein, cells activate a well-orchestrated ensemble of intracellular signal transduction pathways, collectively termed the unfolded protein response (UPR) [24]. We monitored changes of the two principal UPR markers, (PKR)-like ER kinase (PERK) and inositol-requiring gene-1 (IRE1) and their essential downstream effectors in breast cancer cells exposed to NS1643. Splicing of the X-Box Binding Protein-1 (XBP1) mRNA was selected as indicator of UPR early response while Protein Disulfide Isomerase (PDI), DnaJ Heat Shock Protein Family (Hsp40) Member C3 (DNAJ3), C/EBP homologous protein (CHOP), Adaptor Subunit Of ERAD E3 Ubiquitin Ligase (SEL1) were randomly selected from the list of upregulated genes. We found that NS1643 treatment for 24hr produced a significant upregulation of all UPR markers in the TNBC cell line MDA-MB-231 (Fig. 4C; 4D). Nevertheless, these NS1643-driven alterations appeared to be transient as cells treated with NS1643 for 48hr presented a normal level of UPR markers (Supplementary fig. 2 B). Also, to establish whether stimulation of Kv11.1 altered gene transcription independently of breast cancer subtype, we performed validation experiments in the ER + cell line, MC7. We found that these cells respond to the NS1643 treatment similarly to TNBC (Fig. 4E; 4F). In contrast, supported by the lack of Kv11.1 expression in normal breast cells, none of the UPR markers changed significantly in non-carcinogenic MCF10A breast cells treated with NS1643 (Supplementary Fig. 2C). This data suggests that stimulation of the Kv11.1 produces an ER stress/UPR response in breast cancer cells.

### 2.6 Kv11.1 activity produces oxidative stress in breast cancer tumoroids

**Fig. 5:**
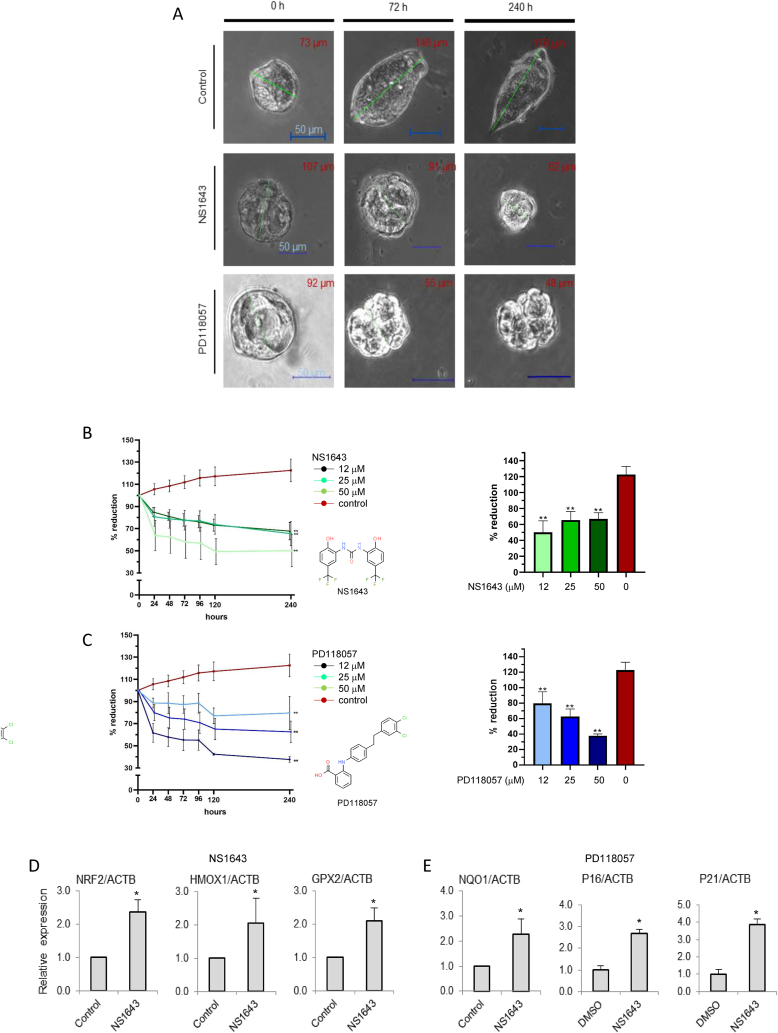
**Activation of Kv11.1 suppresses growth and activates an oxidative stress response in breast cancer tumoroids.** A) Inhibitory effect on representative patient-derived organoids (PDOs) by Kv11.1 stimulation via two chemically distinct activators, NS1634 or PD118057; (n = 3; Data = Mean ± SEM; *<0.05; **<0.0001). Core biopsy specimens were collected from 3 distinct patients diagnosed with TNBC and grown as tumoroids. B) PDOs treated with different concentrations of NS1643 and percent size reduction after 240hr or C) PD118057 and percent size reduction after 240hr. (N = 3 for each treatment; *<0.05; *<0.001; ***<0.0001). C) mRNA expression level of antioxidant genes NRF2, HMOX, GPX2 or E) NQ01 and senescence markers (P16 and P21) in PDOs treated with NS1643 for 2 or 24 h vs control *(n = 3; Data = Mean ± SEM; *<0.05; *<0.001).

To validate the clinical relevance of our findings, we tested the effects of pharmacological stimulation of Kv11.1 on both TNBC (Fig. 5) and ER+ (supplementary Fig. 3A, 3B, 3C) patient-derived organoids (PDO) which recapitulate morphological and genetic features of the original tumor [[25], [26], [27]]. Biopsy cores were obtained from breast cancer patients with no prior neoadjuvant endocrine or cytotoxic therapy following institutional review board (IRB) approval and patient consent. These experiments show that treatment of TNBC PDOs with two distinct Kv11.1 channel activators, NS1643 or PD118057, produced a significant and progressive reduction in the number of tumor cells followed by growth arrest (Fig. 5A; 5B, 5C; Supplementary Fig. 3B, 3D, 3E). Also, antioxidant genes observed in human-derived cell lines were also found to be upregulated in PDOs treated with NS1643 (Fig. 5D) or PD118057 (Fig. 5E) as compared to vehicle treated controls indicating stimulation of Kv11.1 produces oxidative stress in breast cancer PDOs.

We previously demonstrated that stimulation of Kv11.1 in breast cancer cells can activate a senescent-like phenotype which is characterized by cell cycle arrest [18,28]. Therefore, we monitored markers of cellular senescence in PDOs as soon as they reached steady state mass (5 days). We observed that both p21cif and p16ink4 senescent markers were significantly upregulated in PDOs treated with NS1643 when compared with control (Fig. 5E). Similar effects were observed when Kv11.1 activators were applied to ER + breast cancer PDOs (Supplementary Fig. 3D). These results confirm *in vitro* observations in a relevant *ex vivo* model of breast cancer that stimulation of Kv11.1 channel activity produces oxidative stress and senescence [18,28].

### 2.7 NRF2 antioxidant response compensate the lethal effect of Kv11.1 activation

Our experiments revealed that the Kv11.1 activity-dependent ROS production is transient suggesting that cells can activate a redox buffering mechanism.

Nuclear factor E2-related factor 2 (NRF2) is a transcription factor that coordinates the basal and stress-inducible activation of a vast array of antioxidant genes including NQO1, HMOX1, GPX2 and itself (positive feedback).

**Fig. 6:**
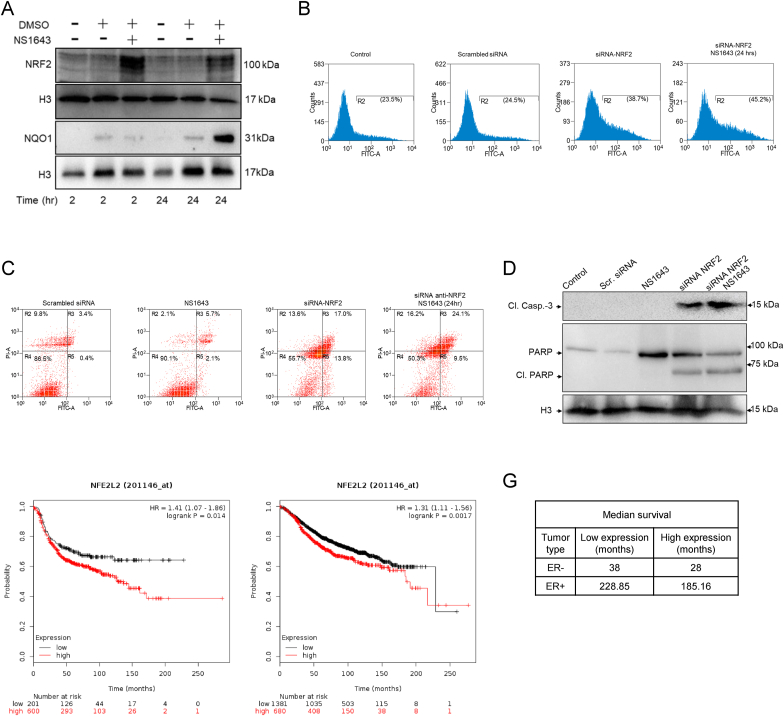
**NRF2 is a critical factor for Kv11.1-dependent stress response.** A) Western blot shows the effect of NS1643 on NRF2 and NQO1 protein expression level in MDA-MB-231 at different time points. B) Flow cytometry was used to measure ROS production in MDA-MB-231 breast cancer cells in which NRF2 has been suppressed by siRNA (siRNA-NRF2). C) Flow cytometry detection of apoptosis via Annexin V staining. D) Western blot analysis of cleaved caspase-3 and PARP in breast cancer cells as in B. E) Kaplan–Meier plots of overall survival in patients with TNBC or F) ER + BC in patients with high (red) and low (black) expression of NFE2L2 gene (top vs. bottom tertiles). G) Median survival in low expression vs high expression cohort. Hazard ratios (HR) compare the hazard of relapse or death in the high expression vs. low expression groups. (For interpretation of the references to color in this figure legend, the reader is referred to the Web version of this article.)

Interestingly, analysis of the time-dependence of the effect of NS1643 on redox markers revealed that NRF2 mRNA (Supplementary Figure 4A) and protein level significantly increased 2hrs after treatment (Fig. 6B) whereas transcription of downstream effectors occurred only after 24hr of NS1643 application, as indicated by the change of NQO1 protein level (Fig. 6A).

To gain insight on the antioxidant role of NRF2 in breast cancer cells treated with NS1643, we measured ROS levels in cells in which NRF2 was suppressed by siRNA (siRNA-NRF2; Supplementary Figure 4B) and then treated with NS1643 (Fig. 6B). Remarkably, in contrast with naïve cells, siRNA-NRF2 cells exposed to NS1643 for 24hr did not show reduced ROS level. Also, NS1643 did not produce cell death in control cells [18,28] but in siRNA-NRF2 cells NS1643 activated apoptosis as indicated by a significant increase of Annexin V expression (Fig. 6C) and cleavage of capsase-3 and PARP1 (Fig. 6D; Supplementary Fig. 4D). Therefore, this data indicates that stimulation of Kv11.1 channel activity produces an alteration in redox homeostasis by upregulating the NRF2 survival factor which can provide a survival mechanism for breast cancers during oxidative stress.

To explore the clinical relevance of our findings, we conducted in silico analysis with the Kaplan-Meier Plotter database (KM plotter.com), which is a curated gene expression database of publicly available microarray datasets that performs survival analyses based on selected biomarker expression levels. This investigation revealed that high expression of the NRF2 gene (NFE2L2) is associated with decreased overall survival (OS) in breast cancer patients independently of the expression of estrogen receptor (Fig. 6E) with a 26% and 20% increase in mortality (Fig. 6F) in TNBC and ER + breast cancer patients, respectively.

## 3 Discussion

Resistance to death is a hallmark of cancer and is dependent on the ability of the cancer cells to adapt to multiple insults. Consequently, cancers can evoke a wide array of responses to therapeutic treatments, thus identification of mechanisms controlling the compensatory response of cancer is fundamental for developing anticancer therapies. Our data suggest that breast cancer cells exposed to Kv11.1 activators undergo an initial oxidative stress to which cells adapt by activating the transcription of antioxidant genes including NRF2.

Analysis of the effects of Kv11.1 activity by using activator molecules (NS1643 or PD115087) show that stimulating Kv11.1 produces mitochondria damage. We have previously demonstrated that Kv11.1 opening causes Ca^2+^ entry in breast cancer cells [19]. As expected, application of the Ca^2+^ chelator EDTA inhibited the NS1643 effect on ROS production (supplementary figure 5). Kv11.1 activity-dependent Ca^2+^ entry can be explained by the increase in negative charge in the intracellular environment produced by the loss of K^+^ (upon Kv11.1 opening) which provides a driving force for Ca^2+^ entry. Consequently, mitochondria undergo Ca^2+^ overload, became damaged and increase ROS production. Remarkably, we have previously demonstrated that cancer cells can activate autophagy as a survival mechanism to NS1643-dependent stress [29] and, it has been extensively demonstrated that cells can activate autophagy to degrade severely damaged mitochondria [30]. Therefore, at this time we can speculate that NS1643-dependent damaged mitochondria are degraded by the autophagy process.

Interestingly, although the Kv11.1 activator was applied chronically, we observed that after an initial increase the ROS concentration was reduced to a level that was lower than control (untreated cells).

The analysis of our microarray assay revealed that chronic stimulation of the Kv11.1 potassium channel alters transcription of many antioxidant ROS scavengers including thioredoxin (Trx), catalase (CAT), superoxide-dismutase (SOD), and the nuclear factor erythroid 2 (NRF2) pathways which include a large group of ARE-regulated genes [[31], [32], [33]].

This event was validated by experiments performed in relevant *in vitro* and *ex vivo* systems which included PDO models of different types of breast cancer (ER+ and TNBC). PDOs represent the front line of pharmacology testing in cancer and have the potential to offer an efficient way to assess complex biology within an appropriate timeframe to allow clinical direction of treatment using precision medicine.

ROS represent a variety of different chemical entities including peroxides, superoxide, hydroxyl radical, singlet oxygen that are all reactive and can disturb a variety of cellular signaling mechanisms. Nevertheless, upregulation of the large group of ARE-regulated antioxidant genes by NS1643 suggests generic buffering events rather than alteration of a specific ROS member-dependent signaling.

Interestingly, NS1643 also activated a UPR response. The endoplasmic reticulum is a highly specialized organelle responsible for multiple cellular functions including protein folding and the maturation and maintenance of cellular homeostasis. Activation of the UPR biochemical cascade is triggered by the accumulation of improperly folded proteins in the ER. Proper protein folding requires mildly but distinct oxidizing and reducing environments (redox) within the endoplasmic reticulum. This suggests that an excessive amount of ROS produced by stimulation of Kv11.1 could unbalance redox in the endoplasmic reticulum, lead to accumulation of unfolded proteins and therefore, activate UPR. Nevertheless, similar to the ROS production, the UPR response markers increased initially (24hr) and then reduced (48hr). This event can be explained by the protective action of ROS scavenger antioxidant genes that reached maximum effect after 24hr of NS1643 treatment. The rescuing activity of the antioxidant genes was confirmed by the dramatic effects produced by suppression of the NRF2 function. Unfortunately, there are no specific or powerful enough NRF2 inhibitors that can significantly limit transcription of ARE-regulated genes. This inefficiency was confirmed by our attempts to use several pharmacological tools including ML-385 at high doses (mM range). Nevertheless, small interference RNA produced a strong suppression of NRF2 expression and a significant downregulation of the ARE-regulated genes in breast cancer cells. In contrast with previous observations, in these cells the application of NS1643 evoked an increase of ROS concentration that was not reduced after 24hr and activated a caspase-dependent death pathway. We concluded that lack of NRF2 sensitized cells to the lethal effect of NS1643 by impairing the cellular ability to buffer ROS.

The role of ROS and/or NRF2 in cancer is still heavily debated as both factors appear to play a major role in all different phases of carcinogenesis. Also, several therapeutic strategies in cancer clinical trials aiming to suppress ROS production have produced disappointing results. These studies employed natural or synthetic antioxidants or NRF2 activator molecules which were ineffective in reducing cancer growth mostly because of their unspecific action and because the ability of cancer cells to adapt to stress. In contrast, inhibition of NRF2 has not been considered thoroughly. In view of our data and on the fact that the intended use of several therapeutic strategy is to cause an increase of ROS (e.g. radiation therapy), we speculate that inhibiting NRF2 would remove an important survival mechanism in cancer cells.

**Fig. 7:**
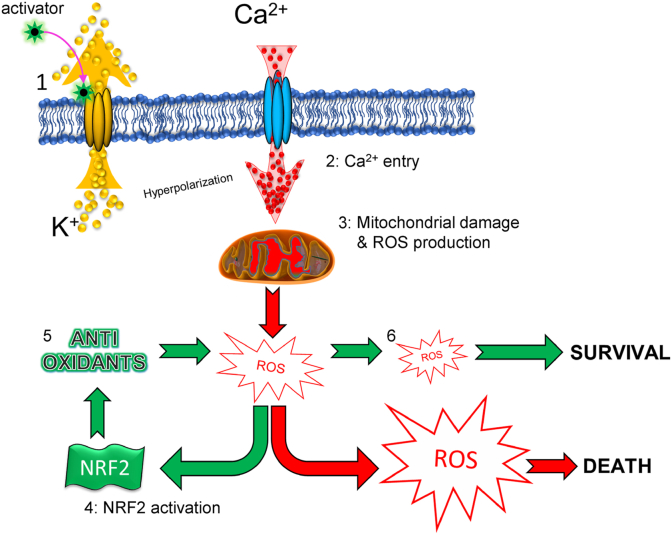
**Schematic representation of the effects of pharmacological stimulation of the Kv11.1 potassium channel on ROS.** 1) Opening of the Kv11.1 channel produces hyperpolarization (K^+^ ion exiting the cell leads to an increased intracellular negative charge) which 2) provides a driving force for Ca^2+^ entry. Consequently, 3) mitochondria are damaged by Ca^2+^ overload which associates with alteration of cellular redox by producing ROS. ROS concentration is buffered by activation of the 4) NRF2-dependent transcription of 5) antioxidant genes which rescues 6) the lethal effect of excessive ROS production.

In conclusion, our work demonstrated that breast cancers activate a compensatory mechanism driven by NRF2 to survive the lethal effect of K^+^ channel activators (Fig. 7).

## 4 Material and methods

### 4.1 RNA-Seq

Quality of the total RNA was measured by the Agilent 2100 Bioanalyzer. Concentration of the input material required for library preparation was determined using the Qubit Fluorometer. Library Preparation & Sequencing: Using the Illumina TruSeq Stranded mRNA Library Prep kit the mRNA in the total RNA was converted into a library of template molecules of known strand origin. Specifically, mRNA molecules were isolated using poly-T oligo attached magnetic beads. The resulting mRNA was fragmented and reverse transcribed using random primers. The cDNA product was then amplified, incorporating sequencing adapters and barcodes to create a final double-stranded cDNA library ready for sequencing. The samples were sequenced on the Illumina HiSeq platform rendering 50 bp single-end reads.

### 4.2 Data Analysis

Adapter sequences were removed and low quality reads were trimmed using Cutadapt (v. 1.11). The resulting reads were mapped to the human reference genome from Ensembl, GRCh38, using Bowtie2 (v. 2.1.0). The aligned sequencing reads and a list of genomic features were used as input for the Python package HTSeq (v. 0.6.1p1) to count the mapped genes and generate a table of raw counts. The DESeq2 package (v. 1.14.1) was used to determine differential expression between sample groups using the raw count table by fitting the negative binomial generalized linear model for each gene and then using the Wald test for significance testing. Count outliers were detected using Cook's distance and were removed from further analysis. The Wald test p-values from the subset of genes that passed an independent filtering step were then adjusted for multiple testing using the Benjamin-Hochburg procedure.

### 4.3 Mitochondrial Morphology

MDA-MB-231 were seeded at 1.5 × 105 cells/well in standard 6-well plates containing coverslip, in DMEM (1.5 mL) for 24 h to ensure attachment. Mitochondria were then stained with 200 nM Mitotracker green for 20 min at 37 °C. After the incubation, compounds were added as indicated in the figure and mitochondria morphology was observed at the indicated time points by fluorescence microscopy using a Leica TCS SP5 Confocal Laser Scanning Microscope (Leica Microsystem).

Cells plated on glass bottom dishes were incubated with 50 nM TMRM for 30 min and then washed with PBS twice. Live cell imaging was performed by a Zeiss LSM880 META confocal microscope equipped with a PeCon heated stage. Individual 8-bit images were acquired by DPSS 561-10 laser with 2% laser power and a PMT detector. The pinhole was set to achieve 1 Airy unit. Cells were treated with 2 μl DMSO or 50 μM NS1643 before image acquiring. To analyze mitochondrial morphology, images were processed with ImageJ and ImagePro plus 6.0. Mitochondria were categorized into different length groups, the short (<1.5 μm), medium (1.5–10 μm) and elongated (>10 μm). Total mitochondrial area and sum area of each group were measured by ImagePro plus. The percentage of each group was calculated by dividing sum area of each group with total mitochondrial area. Fluorescence intensity of TMRM was measured to represent the change of mitochondrial membrane potential.

### 4.4 Oxygen Consumption Assay

Oxygen consumption rate (OCR) was measured by using an XF24 Extracellular Flux Analyzer (Seahorse, Bioscience). MDA-MB-231 and MCF-7 were seeded at 4 × 104 cells/well and 4.5 × 104 cells/well respectively in 100 μl of complete DMEM. 24 h after the seeding, the medium was replaced with 670 μl/well of high-glucose DMEM without serum and sodium bicarbonate and supplemented with 1 mM sodium pyruvate and 4 mM l-glutamine. OCR was measured at preset time intervals upon the preprogrammed additions of the following compounds: NS-1643 at different concentrations as reported in the figure, oligomycin to 2 μg/mL, FCCP to 500 nM for MDA-MB-231 and to 300 nM for MCF-7, antimycin A to 1 μM final concentrations. All chemicals were added in 70 μl of DMEM. At the end of each experiment, cells were observed using an optical microscope to exclude wells with a massive loss of cells because of their death and detachment (not shown).

### 4.5 Cell and PDO culture

Adherent cell lines MCF7, MDA-MB-231 and MCF10A were maintained in DMEM supplemented with 10% newborn calf serum. PDOs: core biopsy specimens were collected from patients that were diagnosed with TNBC and grown as spheroids which were used for RNA isolation and qRT PCR analysis after 10 days in culture.

Collection and preparation of primary human tissue. Upon biopsy arrival tissue was split in half, with one piece fixed in formalin for histopathological analysis the remainder processed for viable tumor cells. The remaining tissue was washed once with PBS and any obvious non-tumor tissue was removed and minced in ~1 mL of AdDF+++ (Sachs et al., 2018) using surgical scalpels until macro-suspensions were observed. The tissue suspension was strained over a 100 μm filter and centrifuged at 500 g × 5 min. The pellet was suspended in a 1:1 ratio with matrigel (Corning 354230) and kept on ice. 40 μL drops of the matrigel-cell suspension was plated onto pre-warmed 24-well tissue culture (Fisher 0720084) plate on cleaned cover glasses (Fisher 12-545-81). Plate was inverted after 2–3 min and was allowed to solidify at 37C for 30 min. After solidification, 500 μL of BC organoid medium (Sachs et al., 2018) was added to each well and plates were transferred to humidified 37C/5% CO2 incubator. Medium was changed every 4 days and organoids were passaged every 1–4 weeks.

Passaging: Organoids were disrupted by mechanical dissociation by pipetting up and down with a P-1000 to disrupt the matrigel. The suspension was transferred to a 15-mL falcon tube and each well was rinsed with ~1 mL of AdDF+++ media. Organoids were centrifuged at 500g for 5 min, supernatant was aspirated, and the organoid pellet was re-suspended in matrigel in a 1:1 ratio and plated in a 24-well plate following the same procedure as described above.

Growth Assay: Organoids were passaged as described above, and allowed to grow for 1–2 weeks. After imaging initial time point (0 h), organoids were treated with 12, 25, and 50 μM of KV11.1 activators NS1643 (Sigma-Aldrich, 0663) and PD-118057 (Sigma-Aldrich, 313674-97-4). Image acquisition was performed at 0, 24, 48, 72, 96, 120, and 240 h of treated and untreated organoids using a Nikon Eclipse Ti inverted microscope equipped with a 10x/0.30 (Plan Fluor) objective. Images were taken of individual organoids on bright-field at their widest focused plane and were measured by their longest diameter using Nikon NIS-Elements AR 4.60.00. Data analysis was performed using GraphPad Prism 8.4.3. Statistical significance was determined using a paired two-tailed *t*-test, analyzed as mean ± SEM. No statistical difference observed in DMSO controls, and were compiled with positive controls.

### 4.6 siRNA

Anti-NRF2 siRNA was obtained from OriGene (OriGene Technologies MD) and used according to manufacturer's instructions

### 4.7 Western blot

Cells were harvested 2–48h after treatment assays were carried out as described [Proc. Natl. Acad. Sci. USA 96 (1999) 4868–4873]. In short, cells were lysed in JLB (50 mM Tris⋅HCl, pH 8/150 mM NaCl/10% glycerol/0.5% Triton X-100) containing a complete protease inhibitor cocktail (Boehringer-Mannheim). Lysis proceeded for 10 min at 4 °C, after which the cellular debris was pelleted by centrifugation at 14 K for 5 min.

Antibodies against the following proteins were purchased from Santa Cruz Biotechnology, INC (NRF2, (Proteintech; 16396-1-AP [34]; NQO1, sc-32793; actin, sc-8432) or Cell Signaling Technology: (IREα, #3294; PDI, #3501; PERK, 5683; H3, 4499).

### 4.8 RT-PCR

Total RNA extraction was done with TRIzol reagent (Life Technologies Corporation, Grand Island, NY). RNA was transcribed into cDNA with SuperScript® III First-Strand Synthesis SuperMix (Life Technologies Corporation, Grand Island, NY) and analyzed with SYBR green (Applied Biosystems, Inc., Grand Island, NY) on the 7500 FAST Real Time PCR detection system (Applied Biosystems, Inc., Grand Island, NY). The human primers used are: ACTB, forward: 5-ggacttcgagcaagagatgg-3′, reverse: 5′-agcactgtgttggcgtacag-3’; XBP1_S, forward: 5′- ctgagtccgcagcaggtg -3′, reverse: 5′- ggctggtaaggaactgggtc -3′; DNAJC3, forward: 5′- actgaggcctgagcgaga -3′, reverse: 5′- gcaggaaggggaataccgag -3′; CHOP, forward: 5′- agaaccaggaaacggaaacaga -3′, reverse: 5′- tctccttcatgcgctgcttt -3′; SEL1L, forward: 5′- gagaatacggctgcctgatgaag -3′, reverse: 5′- caggtgcagttgtccaagacca -3′; HMOX1, forward: 5′- ccaggcagagaatgctgagttc -3′, reverse: 5′- aagactgggctctccttgttgc -3′; GPX2, forward: 5′- acttcacccagctcaacgag -3′, reverse: 5′- atgctcgttctgcccattca -3′; NQO1, forward: 5′- cctgccattctgaaaggctggt -3′, reverse: 5′- gtggtgatggaaagcactgcct -3′; NRF2, forward: 5′- cacatccagtcagaaaccagtgg -3′, reverse: 5′- ggaatgtctgcgccaaaagctg -3′; P16, forward: 5′- ctcgtgctgatgctactgagga -3′, reverse: 5′- ggtcggcgcagttgggctcc -3′; P21, forward: 5′- aggtggacctggagactctcag -3′, reverse: 5′- tcctcttggagaagatcagccg -3′;

### 4.9 Flow cytometry

For apoptosis analysis, cells were stained with FITC-Annexin V and PI (BD Bioscience, Franklin Lakes, NJ) following the manufacturer's instructions.

For ROS analysis, single-cell suspensions were treated with 20 μM DCFDA and the fluorescence (Ex/Em = 485/535 nm) was measured. Flow cytometry was performed at the University of Illinois at Chicago RRC facility using CyAn flow cytometer (Beckman Coulter Inc., Fullerton, CA). All data were analyzed by Summit software (Beckman Coulter Inc., Fullerton, CA).

## Funding

This project is supported by the

- Department of Defense towards this research is thereby acknowledge (US DOD CDRM W81XWH-20-1-046).
- Christl Burgess Memorial Fund for ovarian cancer research.

## Author contributions

VS, NE, JY helped in performing all *in vitro* experiments including WB, FACS, RT-qPCR. LL and RP helped in performing Sea horse analysis. RGV, RS, helped in performing experiments with PDOs. LL, MB, RM, SG helped in providing conceptual and writing assistance.

## Declaration of competing interest

The authors declare no conflict of interest.
