# Supplementary material for: Compensatory expression of NRF2-dependent antioxidant genes is required to overcome the lethal effects of Kv11.1 activation in breast cancer cells and PDOs
Source: Redox Biol. 2021 Jun 12;45:102030. doi: 10.1016/j.redox.2021.102030 (PMC8220394; doi:10.1016/j.redox.2021.102030)

Supplementary Figure 1

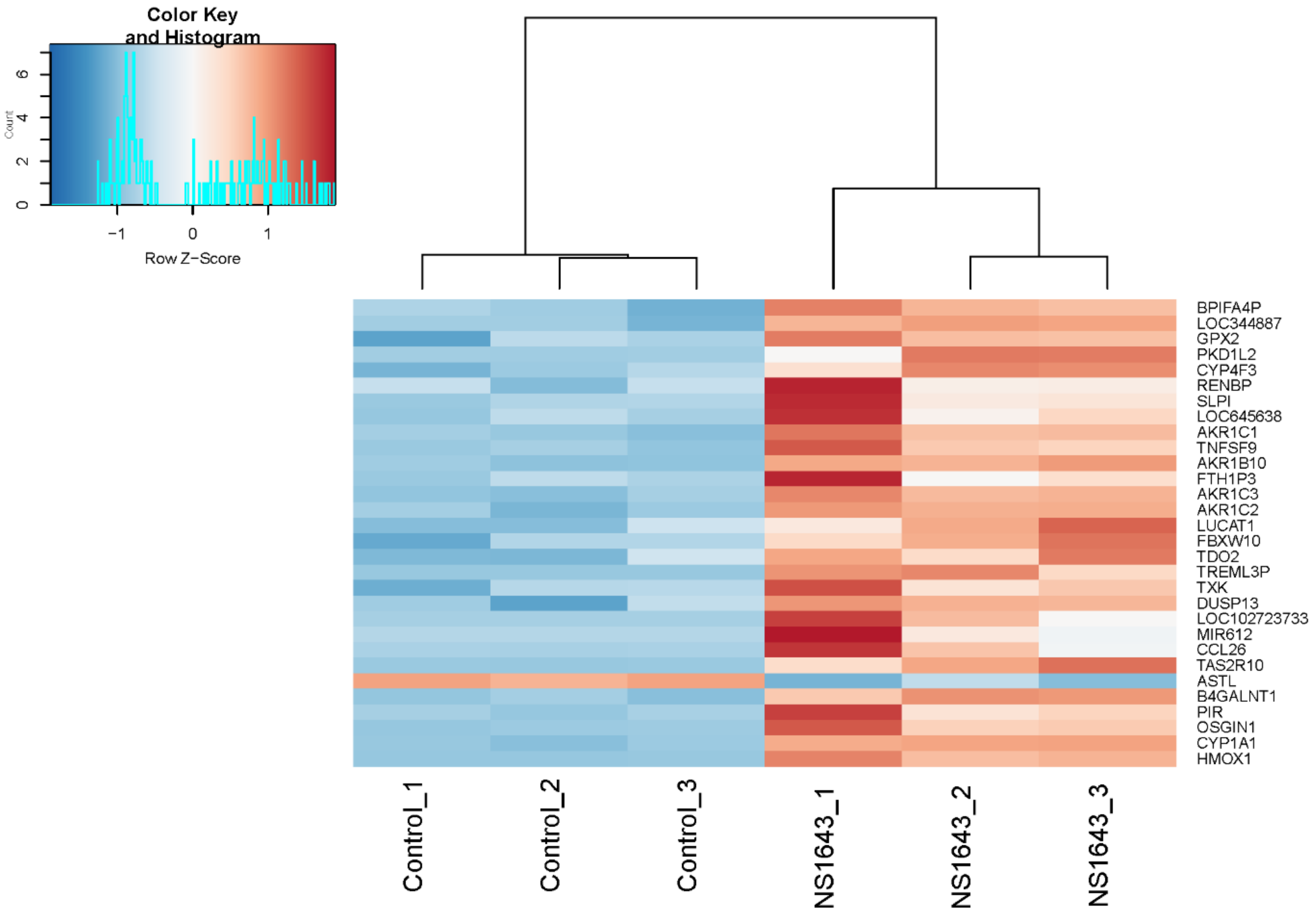

Supplementary Figure 2

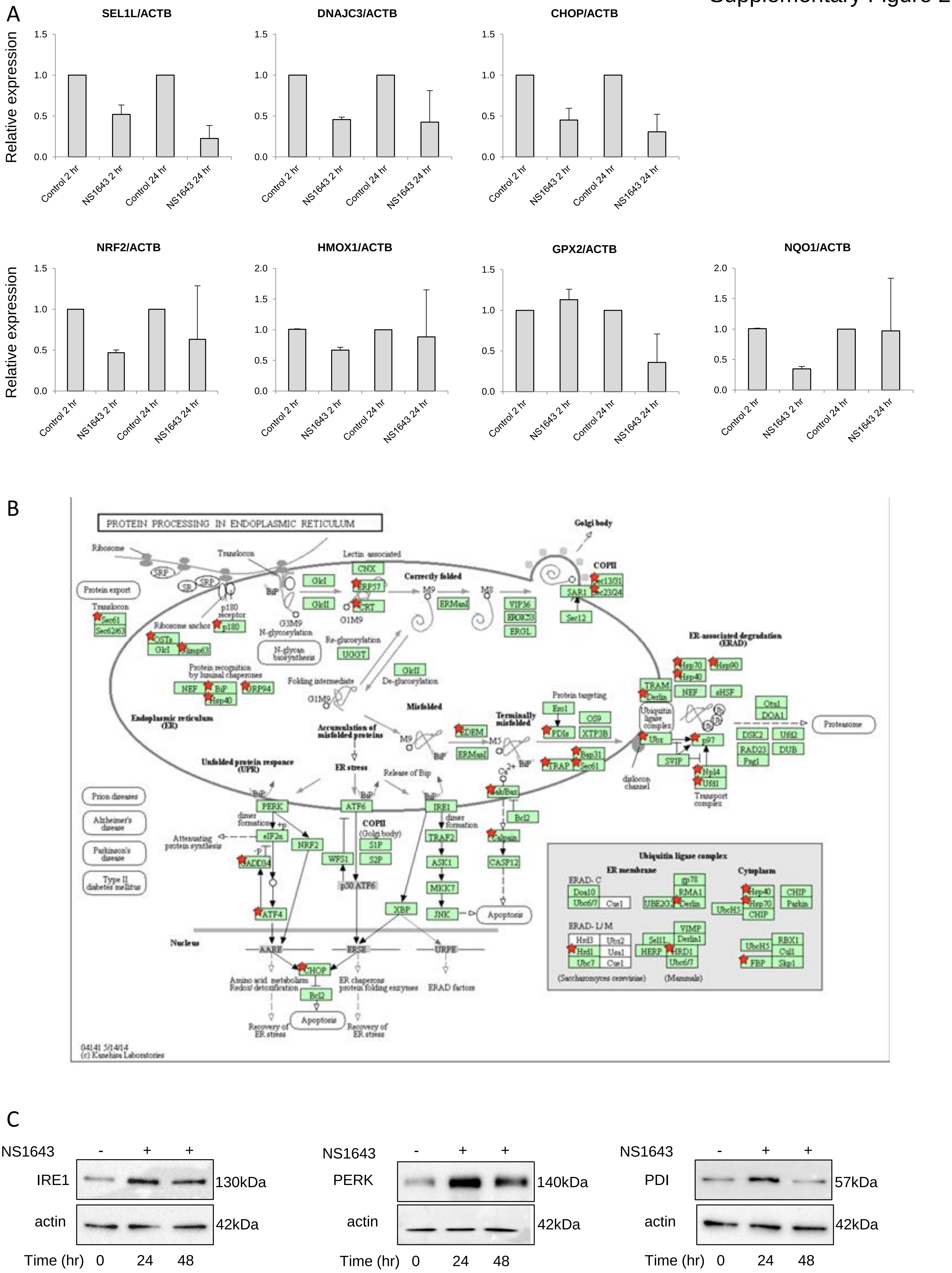

Supplementary Figure 3

A

Patient	Subtype	KI 67%	Diagnosis	Grade	Age		Key	
56	TNBC	70%	IDC	2	44		TNBC	Triple Negative Breast Cancer
57	TNBC	70%	IDC	2	80		ERPR	Estrogen Receptor and Progesterone Receptor Positive
60	TNBC	60%	IDC	3	36		IDC	Invasive Ductal Carcinoa
75	ERPR	10-20%	IDC	2	35			

B

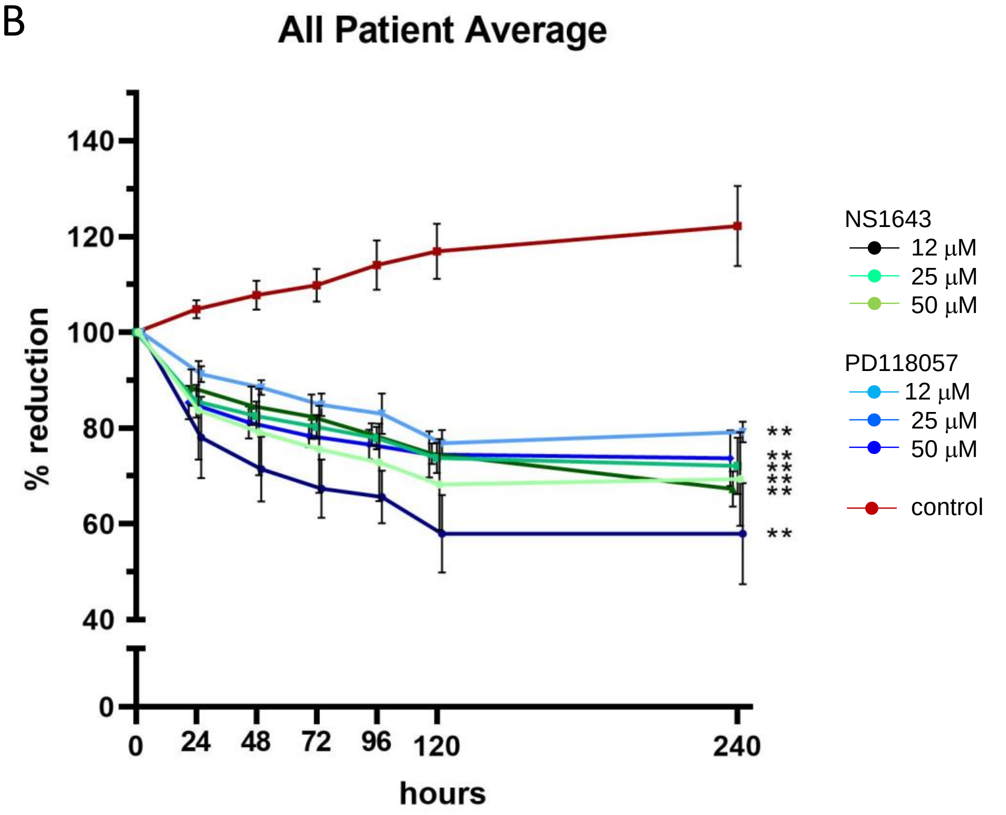

C

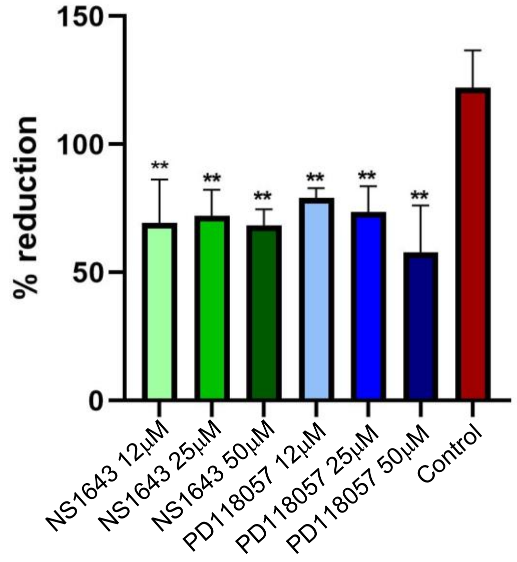

D

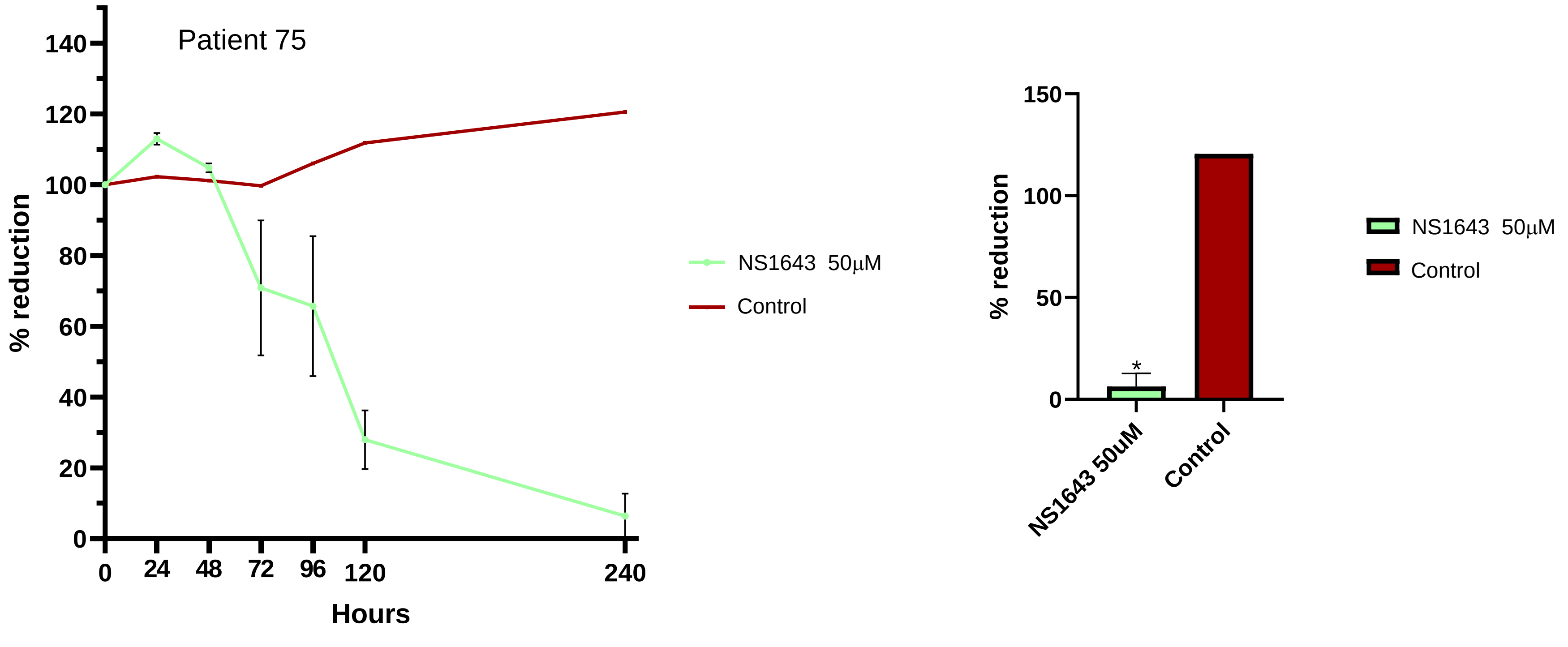

Supplemental Fig. 4

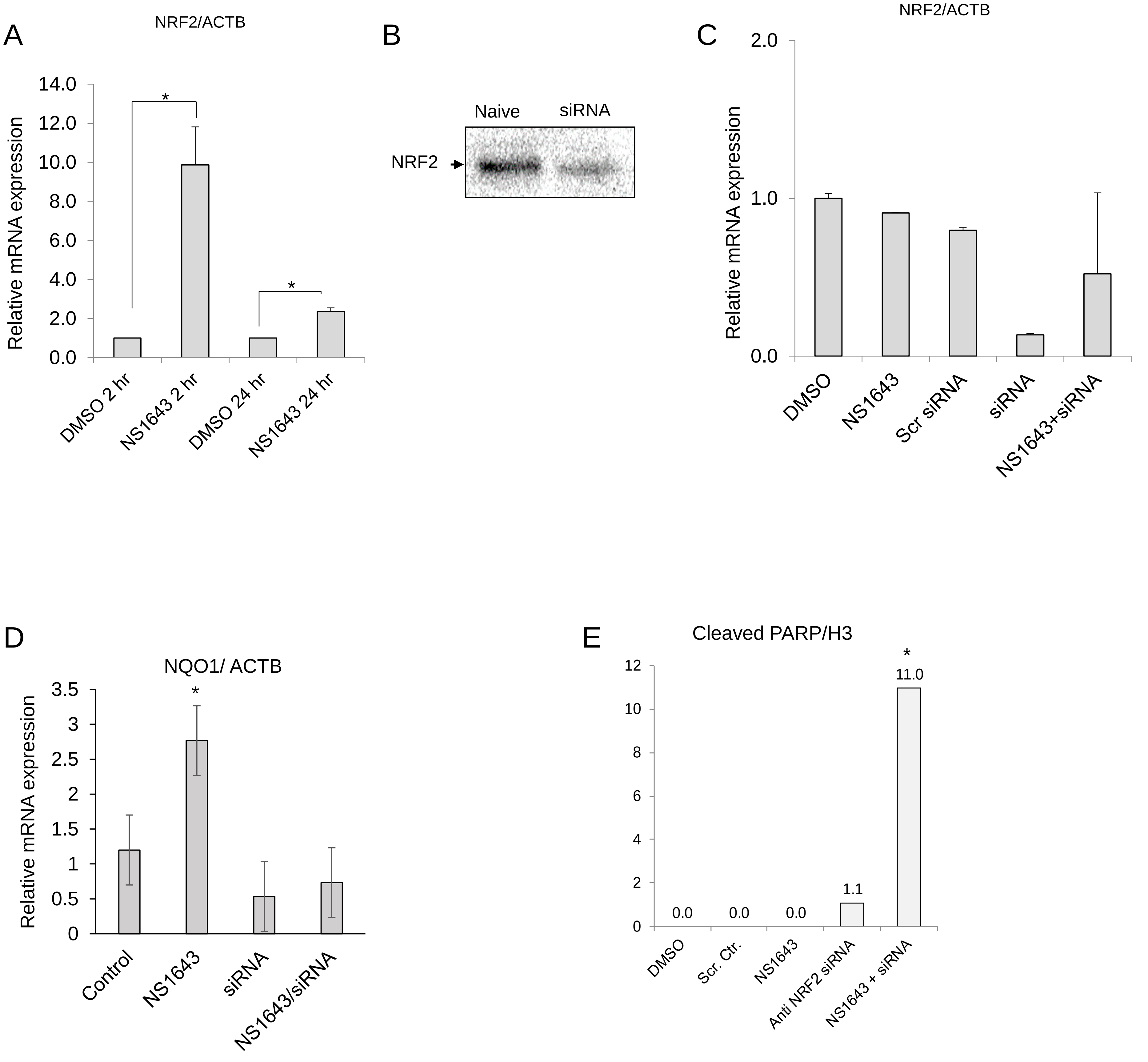

Supplemental Fig. 5

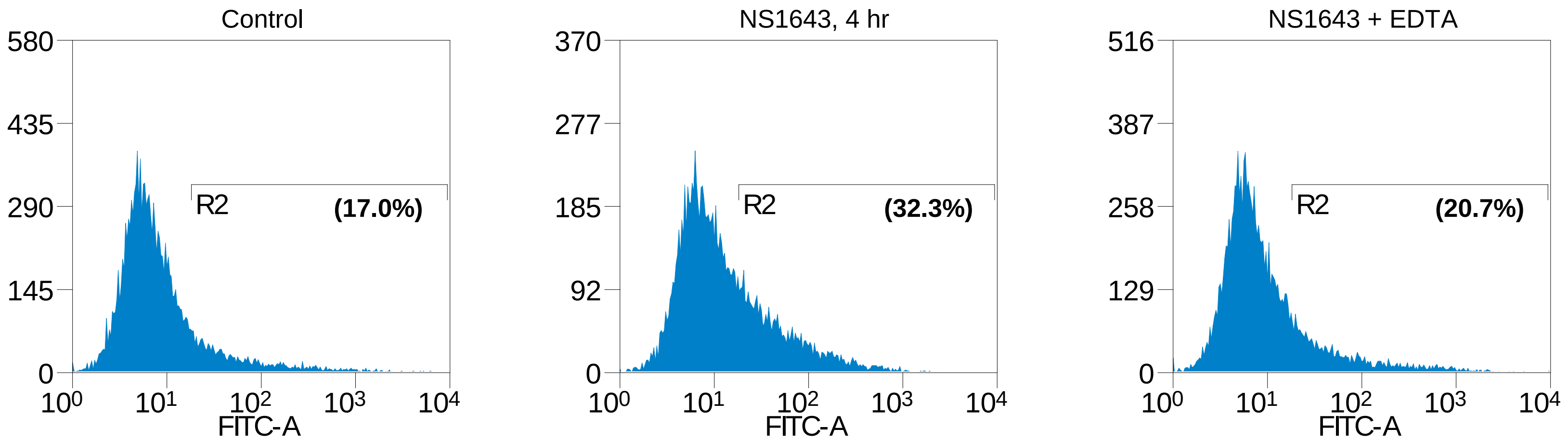

Supplement: Multimedia component 1 [file mmc1.pdf]
